# Narrative of the Poisoning of One Hundred and Eighty Persons, by the Berries of Belladonna

**Published:** 1814-11

**Authors:** M. Gaultier


*For the Medical and Physical Journal.*

### *Narrative of the Poisoning of One Hundred and Eighty Persons, by the Berries of Belladonna;* by M. Gaultier.

On the 14th of September, 1813, a detachment of some hundreds of men of the 12th regiment of infantry, arrived at a hill where unfortunately a group of plants grew, since recognised to be the Atropa Belladonna. Thirsty from a long march, the soldiers fell upon these plants, and soon stripped them of their fruits, some of which were of a deep red colour, whilst others had a violet tint, which was an indication of their ripeness. In vain some persons, who knew the danger of eating them, dissuaded them from it. Some took six or eight, others twelve, others fifty, and some still more. In two hours time the regiment quitted this position, but already 160 of these wretched beings experienced the deleterious effect of the poison. Some expired on the spot where they were gathered, others a little distance from it; others were dragged by their comrades into the neighbouring woods, or wandered there themselves. It was then two o'clock in the afternoon.

Our division, though but a little way off, was not acquainted with this event until the following morning at day-break. A number of the men who were poisoned came to our bivouac, where it was observed they evinced a degree of idiotism; many others were brought in by our patroles. On the evening of the 15th I saw fifteen of them brought in by an officer from the wood. On the 16th, at noon, I saw thirty more, who returned tolerably well, and among them two only could give me an account of the symptoms they had experienced. The first was a young soldier, who had eaten ten or twelve berries: he said he had, in two hours after, such a confusion of sight, that all objects appeared to him to be covered with hay. He could not keep himself erect, but fell to the ground, and as fast as he rose he fell again. He felt a sinking at the stomach, with nausea. The lips, tongue, and palate, were parched. He could not swallow the little particle of saliva that moistened his mouth. It appeared to him that the sides of his throat were in contact, and that he was going to die. Soon every thing appeared to go round him, and he thought he saw no objects but through a thick cloud; then he passed four or five hours in a manner of which he can give no account. The exertion he made, with the assistance of his comrades, brought him gradually about. I saw him forty hours after the accident in perfect health, during which time he had no evacuation from the bowels, which could be considered the consequence of, or operate as a crisis of the accident.

The second patient I interrogated was a sergeant, about forty years old. At first he attempted to dissuade the others from eating, but at length followed their example. He took himself a dozen berries. He fell upon his knees in the ranks, rose, and fell again several times. He had nausea, as in the foregoing instance; his head seemed not to fit his shoulders. Remembering then that he was poisoned, he took, though not without difficulty, some bread, and four green and very sour apples. An hour after, he drank a tumbler of milk. He was soon relieved, and, on the following day, nothing remained but a recollection of the danger he had undergone.

The other soldiers who were brought after their recovery, were stupid, debilitated, and had no recollection of the accident: they had all been deprived of their reason. As to the sixty, and some we had occasion to bring in, they passed the first night in the wood in the middle of a marsh, during a very cold and moist season. Many among them passed a second night there. These unfortunate wretches were almost all bare-headed, without shoes or coat, with their pantaloons about their heels as if they had been to stool; but there was no appearance pearance of fæces on the shirts or pantaloons. All without exception had haggard and staring eyes, suffused conjunctiva, the pupil dilated to a very great degree and immoveable. Their vision was confused, and gave them a false idea of objects. They were in continual agitation. Their knees sunk under the weight of the body, inclining them forwards, and carrying their trembling hands towards the earth, endeavoured to collect little stones and bits of wood, which they always let fall or threw away, to recommence the same pursuit.

The pulse, which I observed only in some, was small, weak, and rather slow than accelerated; but I place no dependance on this symptom, as it occurred in subjects who had been much debilitated by an abstinence of thirty-six hours, with cold and rain, to which they had been exposed naked. Almost all carried on their faces, heads, hands, and arms, the bloody marks of their rencounter with trees, thorns, and rocks, among which they had been drawn, or had fallen; many of those who passed the nights of the 15th and 16th, made a constant noise, crying out at every moment *to arms!* spoke of the Cossacks, &c. and, perceiving our fires through the trees, came bruised and torn upon our advanced guard, and were with great difficulty prevented from throwing themselves into the flames. The symptoms have been so similar in all the cases, that their enumeration will be sufficient to establish the pathognomonic character of of the effect of this poison. In recapitulation we may mention the following: dilatation and immoveableness of the pupil; almost absolute insensibility of the eye to the presence of external objects, or at least confused vision; suffusion of the conjunctiva; prominence of the eyes, which in some are dull, in others shining, ardent, and furious; dryness of the lips, tongue, palate, and throat; deglutition difficult or impossible; nausea, not followed by vomiting; debility; lipothymia; difficulty or impossibility of keeping the erect position; frequent bending of the body forward; continual motion of the hands and fingers; lively delirium, with empty grins; loss of voice, or confused sounds uttered with difficulty, probably tenesmus; gradual recovery of health, without recollection of the preceding circumstances.

This unfortunate event somewhat resembles that which happened near two thousand years ago to the army of ten thousand. When it returned from the expedition undertaken by the young Cyrus against Artaxerxes, and approached Trezibonda, a strange accident befel them, which caused a great consternation among the troops, and is thus described by Xenophon:—As there were many bee-hives in the place, the the soldiers began to eat the honey; after which they were seized with a purging upwards and downwards, followed by delirium; the least affected resembled drunken persons, whilst others were quite furious, and appeared in dying circumstances. The earth was strewed with bodies, as after a defeat; none, however, died: the symptoms ceased on the following day, the men being somewhat weakened by it. Diodorus Siculus relates the same fact, but neither he nor Xenophon give any account of the plant which gave this deleterious impregnation to the honey.

Dioscorides and Pliny have spoken of a poisoned honey found near Heraclea Pontis. Those who eat of it were driven mad. Dioscorides makes no mention of the suspected plant; but Pliny says the honey is gathered from a plant named Ægolethron: and a little farther he observes, the bees collect it from the flowers of Rhododendron.

Tournefort having found the Azalée Pontique and the Rhododendron in great quantity near Trezibonda, has discovered that the honey which poisoned the ten thousand could be nothing but that gathered from the flowers of these plants, and particularly from the former.

